# Field Effect Transistor Biosensor Using Antigen Binding Fragment for Detecting Tumor Marker in Human Serum

**DOI:** 10.3390/ma7042490

**Published:** 2014-03-27

**Authors:** Shanshan Cheng, Kaori Hotani, Sho Hideshima, Shigeki Kuroiwa, Takuya Nakanishi, Masahiro Hashimoto, Yasuro Mori, Tetsuya Osaka

**Affiliations:** 1 Graduate School of Advanced Science and Engineering, Waseda University, 3-4-1, Okubo, Shinjuku-ku, Tokyo 169-8555, Japan; E-Mails: chengshanshan@akane.waseda.jp (S.C.); 1f.sunf@ruri.waseda.jp (K.H.); 2 Institute for Nanoscience & Nanotechnology, Waseda University, 513 Wasedatsurumaki-cho, Shinjuku-ku, Tokyo 162-0041, Japan; E-Mails: hideshima@aoni.waseda.jp (S.H.); skuroiwa@aoni.waseda.jp (S.K.); takn@aoni.waseda.jp (T.N.); prof.hashimoto@aoni.waseda.jp (M.H.); yasurou@aoni.waseda.jp (Y.M.)

**Keywords:** antigen binding fragment, label-free detection, field effect transistor, immunosensor, Langmuir-type adsorption model

## Abstract

Detection of tumor markers is important for cancer diagnosis. Field-effect transistors (FETs) are a promising method for the label-free detection of trace amounts of biomolecules. However, detection of electrically charged proteins using antibody-immobilized FETs is limited by ionic screening by the large probe molecules adsorbed to the transistor gate surface, reducing sensor responsiveness. Here, we investigated the effect of probe molecule size on the detection of a tumor marker, α-fetoprotein (AFP) using a FET biosensor. We demonstrated that the small receptor antigen binding fragment (Fab), immobilized on a sensing surface as small as 2–3 nm, offers a higher degree of sensitivity and a wider concentration range (100 pg/mL–1 μg/mL) for the FET detection of AFP in buffer solution, compared to the whole antibody. Therefore, the use of a small Fab probe molecule instead of a whole antibody is shown to be effective for improving the sensitivity of AFP detection in FET biosensors. Furthermore, we also demonstrated that a Fab-immobilized FET subjected to a blocking treatment, to avoid non-specific interactions, could sensitively and selectively detect AFP in human serum.

## 1. Introduction

Early detection of tumor markers is critical for the survival of cancer patients [1–4]. A variety of technologies have been developed for biomarker detection, such as the enzyme-linked immunosorbent assay (ELISA). However, many of these methods feature complicated and time-consuming labeling processes. Label-free detection for medical diagnosis has attracted much attention, and detection based on field-effect transistor (FET) biosensors may have advantages in terms of their rapidity and sensitivity [5–11]. However, the detection of proteins at low concentrations using FET biosensors is hampered by charge screening effects. Especially in clinical conditions, contaminating proteins such as human serum albumin (HSA) in blood serum might prevent the target proteins from specifically adsorbing onto the sensing surface, suggesting that the sensitivity needs to be improved. To enhance the sensitivity, the use of small receptors enables a binding reaction to occur within the Debye length, which is related to the detection range of the charged target protein in solution. This results in an enhancement of sensitivity and lower detection limits of the sensing system. Many approaches to enhance sensitivity are based on the use of small molecules, such as glycan, antigens, aptamers, polypeptides, and aromatic compounds [12–16]. An alternative, efficient approach is to use an antigen binding fragment (Fab) [17–19]. A Fab is capable of recognizing a target molecule with high binding affinity and specificity. Because the vertical length of a Fab is approximately 6 nm [20], which is smaller than the whole antibody, Fabs can recognize and bind target antigens near the FET surface. In this work, we demonstrate the use of a Fab as a receptor in a FET biosensor. We compared the response of Fab-immobilized, and antibody-immobilized FET biosensors on protein detection and considered the adsorption mechanism of proteins onto the Fab-immobilized surface. Furthermore, to determine effective blocking reagents for the Fab-immobilized surface, we evaluated the surface blocking effects of bovine serum albumin (BSA) and ethanolamine (EA). We also explored the possibility of the quantitative detection of a liver cancer tumor marker, α-fetoprotein (AFP), and performed a series of electrical experiments under various concentrations of AFP in human serum using the Fab-immobilized FET.

## 2. Results and Discussion

### 2.1. Analyses of the Surface of Fab-Immobilized Field-Effect Transistor (FET)

**Figure 1.**
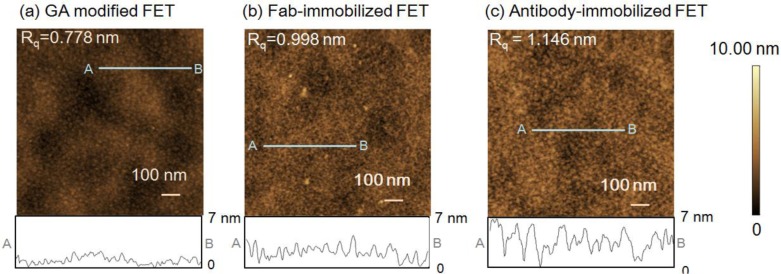
Atomic force microscope (AFM) images of the field-effect transistor (FET) gate surface obtained after (**a**) introduction of glutaraldehyde (GA); (**b**) immobilization of Fab; and (**c**) immobilization of whole antibody. *Z* range = 10 nm. Each image has a cross-sectional profile shown.

First, atomic force microscope (AFM) measurements of the FET sensing surface were performed to examine the height of the immobilized Fab molecules and the change in surface morphology following Fab immobilization. The gate surface of the glutaraldehyde (GA)-modified FET (Figure 1a), 0.778 nm, and Fab-immobilized FET (Figure 1b), 0.998 nm showed differences in the root mean square roughness (*R*_q_). The increase in the *R*_q_ value from the introduction of Fab molecules suggests that Fab molecules were immobilized on the GA-modified FET gate surface. The height of the immobilized Fab surface was 2–3 nm, which was in good agreement with the size of Fab [19]. However, as shown in Figure 1c, the *R*_q_ value for the antibody-immobilized surface was greater than that of the Fab-immobilized surface. In line with our previous reports [9], the height of the immobilized antibodies was measured as 4–5 nm. Thus, the Fab molecules were smaller than the antibodies, and were believed to lie flat on the gate surface.

To check the density of the immobilized receptors, we performed fluorescence measurements (Typhoon 9410, GE Healthcare Bio-Sciences KK, Piscataway, NJ, USA). The density of the immobilized Fab was estimated as 1.23 × 10^4^ molecules/μm^2^, while that of the immobilized antibody was calculated as 0.65 × 10^4^ molecules/μm^2^ in a previous study [10]. Because a single antibody features two antigen-binding sites, the binding sites per unit area was estimated as 1.23 × 10^4^ sites/μm^2^ for the Fab-immobilized surface and 1.30 × 10^4^ sites/μm^2^ for the antibody-immobilized surface, suggesting that both the Fab-immobilized FET and antibody-immobilized FET have approximately the same number of binding sites on the sensing surface in this work.

### 2.2. Comparison of the Sensitivity for Protein Detection between Fab-Immobilized FET and Antibody-Immobilized FET

**Figure 2.**
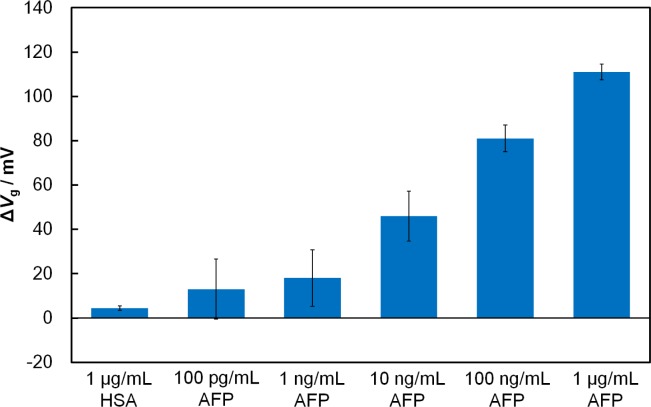
Relation between α-fetoprotein (AFP) concentration and Δ*V*_g_ magnitude for Fab-immobilized FET.

To examine the effect of the receptor’s size, we compared the responses of the Fab-immobilized and antibody-immobilized FETs in the presence of the target tumor-marker protein, AFP. When AFP (1 μg/mL) was added onto the Fab-immobilized FET gate surface to interact with Fab, the threshold voltage shifted (Δ*V*_g_) in a positive direction by 111 mV, while the antibody-immobilized FET had a Δ*V*_g_ of 51 mV. It is clearly evident that the use of the Fab surface immobilization gave a greater FET response to AFP than the antibody immobilization. This can be attributed to the closer approach of AFP to the charge-detectable region of the FET gate surface, which is defined by the Debye length of the protein in solution. Because the Debye length at the gate/solution interface is 7.5 nm in 0.01 × PBS (pH 7.4), part of the bound AFP molecule with a of size 5 nm × 5 nm × 5 nm [21] may remain outside charge-detectable-region in the case of the antibody-immobilized FET. Conversely, with the Fab-immobilized FET, a binding event between Fab and AFP is expected to occur within the Debye length where charge screening effects are minimal. As mentioned in Section 2.1, the number of the binding sites for the Fab-immobilized surface and antibody-immobilized surface were similar, suggesting that the improvement in the sensitivity of the FET-based biosensor in this study results from the reduced receptor size, rather than differences in the number of binding sites. In terms of specificity, the Fab-immobilized FET, showed essentially no shift when a non-related protein, human serum albumin (HSA) (isoelectric point = 4.7) [22], was added instead of AFP. To determine the sensitivity of the Fab-immobilized FET, we examined the response of the FET biosensor to AFP solutions ranging from 100 pg/mL to 1 μg/mL (Figure 2). Increasing the concentration of AFP increased the amount of negatively charged AFP molecules within the Debye length, resulting in a greater shift of Δ*V*_g_. In our previous study [9], the detection limit for AFP of our antibody-immobilized FET was found to be 10 ng/mL. The use of the smaller receptor Fab increased the sensitivity by lowering the detection limit from 10 ng/mL to 100 pg/mL.

**Figure 3.**
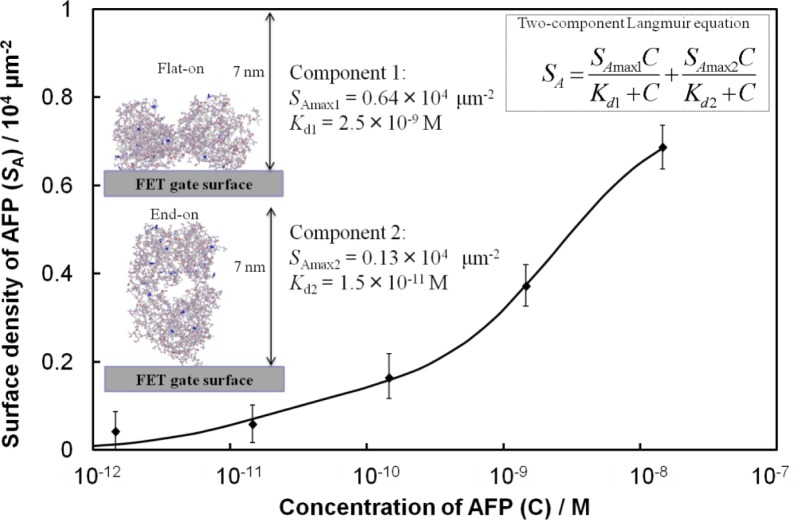
Surface density of AFP, calculated from the magnitude of Δ*V*_g_ and the charge of AFP (see text), as a function of AFP concentration. Inset shows the schematic representation of the two different orientations of Fab on surfaces and the two-component Langmuir-type adsorption model equation.

From Figure 2, we consider an analytical model for the Fab-immobilized FET biosensor response. The relationship between Δ*V*_g_ and AFP concentration is nonlinear, therefore, a calibration scheme needs to be developed based on the adsorption mechanism to compare the sensing results across FETs. We estimated the surface density (*S*_A_) of AFP molecules from the surface charge density (σ_0_) and the charge of AFP. The value σ_0_ was calculated from the change in the surface potential by setting the Stern potential in the Grahame equation [8] equal to Δ*V*_g_. The concentration-dependent adsorption density of the immobilized AFP molecules on the surface was in good quantitative agreement with a model based on the Langmuir adsorption isotherm for equilibrium protein binding, as shown in Figure 3, where the best fit to the data yielded a two-component Langmuir equation. Here, the values of *S*_Amax_ and the dissociation constant (*K*_d_) represent the maximum surface density and affinity properties of the biomolecule interactions, respectively, for each component. Component 1 exhibits a low affinity for AFP (*K*_d_ = 2.5 × 10^−9^ M), while component 2 shows a stronger affinity (*K*_d_ = 1.5 × 10^−11^ M). We assume that the difference in affinity arises from different orientations of the Fab molecules. Component 2 may be related to Fab molecules that are vertically immobilized on the surface (*i.e.*, end-on orientation), towards which AFP may approach more easily with reduced steric hindrance. Conversely, component 1 may be related to Fab molecules lying flat on the surface that may be expected to show lower affinity. Considering the value of *S*_Amax_ for each component, the number of AFP molecules binding to Fab in a flat-on orientation is greater than that of the end-on oriented Fab.

### 2.3. Treatment for the Blocking of Nonspecific Adsorption

**Figure 4.**
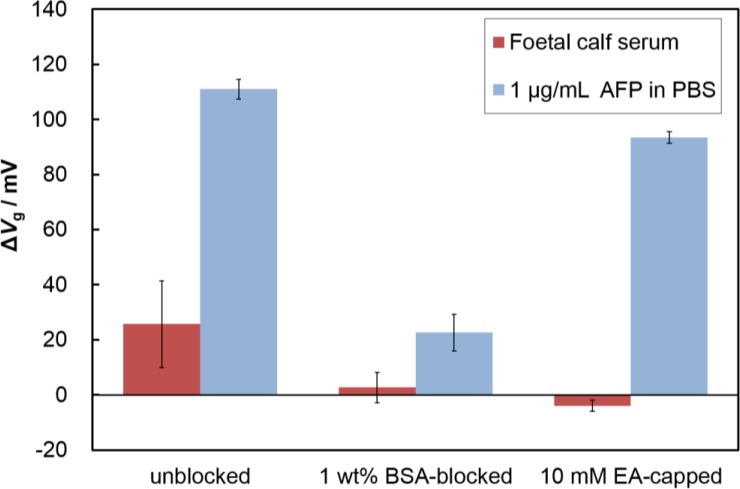
Comparison of sensor response to protein addition for antibody-immobilized FET (non-blocked, bovine serum albumin (BSA)-blocked, and ethanolamine-capped). The proteins were foetal calf serum for negative control and AFP (1 μg/mL) for positive control.

Nonspecific adsorption limits the sensitivity of FETs when used for the detection of specific analytes in serum; therefore, a blocking treatment is necessary to reduce nonspecific protein adsorption. To find an effective blocking reagent for the Fab-immobilized surface, we evaluated the surface blocking effects of BSA and ethanolamine (EA). We immobilized Fabs on the surface, and introduced the blocking reagents (1 wt% BSA or 10 mM EA). As shown in Figure 4, the blocking treatment using BSA or ethanolamine (EA) solution both suppressed nonspecific adsorption. The EA-capped Fab-immobilized surfaces maintained the same response to the addition of AFP as the non-blocked Fab-immobilized surface, while the BSA-blocked, Fab immobilized surface showed a lower response than that of the specific adsorption experiments. BSA may hinder the attachment of target proteins because of its size (3.5 nm × 7 nm × 7 nm) [23], causing Fab binding sites to become obstructed by BSA molecules. In contrast, the Fab-immobilized surface capped with EA maintained its binding capacity for the target protein. Thus, we selected the EA treatment as an effective blocking method for our Fab-immobilized FET.

### 2.4. Quantitative Detection of AFP in Human Serum Using Fab-immobilized FET

**Figure 5.**
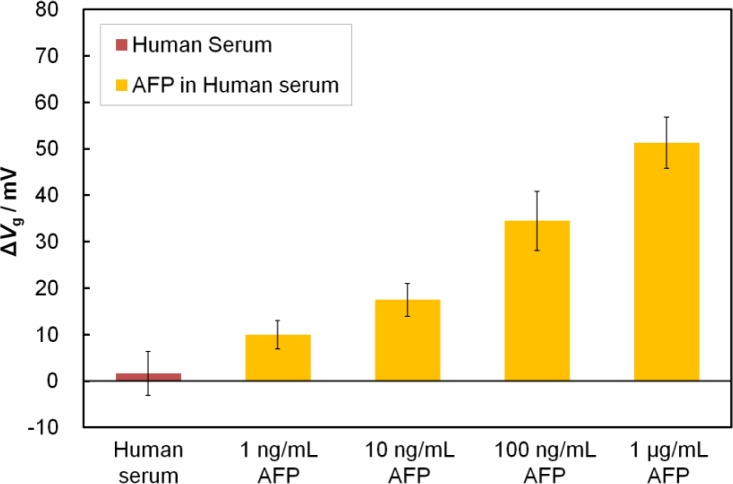
Detection of AFP in human serum by using Fab-immobilized FET with ethanolamine-capping treatment. The concentrations of AFP ranged from 1 ng/mL to 1 μg/mL.

After solving the nonspecific adsorption problem, we investigated the quantitative detection of AFP in human serum. We demonstrated that the Fab-immobilized FET detected AFP contained in human serum at varying AFP concentrations ranging from 1 ng/mL to 1 μg/mL (Figure 5). This range covers most of the clinically relevant concentrations. It should be noted that the magnitude of Δ*V*_g_ at 1 ng/mL AFP in human serum was equal to that at 100 pg/mL in buffer solution, suggesting that other protein contaminates in the serum may obstruct the adsorption of AFP to the sensor surface. However, even under such unfavorable conditions, the EA-capped Fab-immobilized FET achieved detection of AFP at the low level of 1 ng/mL (signal-to-noise ratio >3), which is below the cut-off value for normal levels in humans (<10 ng/mL) [24]. Additionally, detection was completed within 60 min, which is much quicker than using a commercialized ELISA kit that usually takes approximately 3 h from serum sample incubation to the final test results. For the clinical application of FET biosensors in the future, the separation and concentration devices may be combined with our FET sensing device to sensitively and specifically detect the target molecules in blood samples [25].

## 3. Experimental Section

### 3.1. Materials

The antigen, human AFP was purchased from MP Biomedicals, LLC (Santa Ana, CA, USA). Monoclonal anti-AFP (human) was purchased from Nippon Bio-test Laboratories Inc. (Tokyo, Japan). The Fab Preparation kit was purchased from Thermo Scientific Inc. (Waltham, MA, USA). Foetal calf serum was purchased from A&E Scientific (Hainaut, Belgium). Human serum and a self-assembled monolayer reagent, 3-aminopropyltriethoxysilane (APTES) were purchased from Sigma–Aldrich Inc. (St. Louis, MO, USA). BSA was purchased from Jackson ImmunoResearch Laboratories Inc. (West Grove, PA, USA), and all other chemicals were purchased from Kanto Chemical Co. Inc. (Tokyo, Japan). Fab was prepared by using the Fab Preparation kit according to the manufacturer’s instructions. The proteins were used without further purification. PBS (pH 7.4) was made in the laboratory and was prepared using 137 mM NaCl, 8.1 mM Na_2_HPO_4_·12H_2_O, 2.7 mM KCl, and 1.5 mM KH_2_PO_4_. Diluted PBS, 0.01 × PBS (pH 7.4), was prepared by diluting 1 × PBS with ultrapure water.

### 3.2. Detailed Information for the Field Effect Transistor (FET) Device

**Figure 6.**
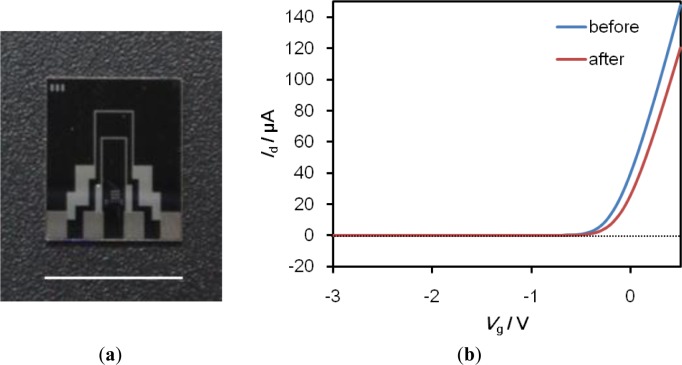
(**a**) Optical microscopic image of the FET device fabricated through the semiconductor processes. The bar indicates 10 mm; (**b**) Change in the *V*_g_-*I*_d_ characteristics of FET device before and after the addition of the proteins.

We have successfully developed SiO_2_-gate FETs with a high degree of chemical durability through surface modification technology using a self-assembled monolayer (SAM) [5], and have successfully engaged in the technology transfer of the fabrication process to Toppan Printing Co., Ltd. (Tokyo, Japan). A photograph of the FET device is shown in Figure 6. The gate size of the FET device is 10 μm (length) × 1000 μm (width). The FET detects potential changes on its gate surface in terms of the intrinsic charge of target proteins that bind specifically to probe molecules immobilized on the gate surface, such as the threshold voltage shift (Δ*V*_g_), or the gate voltage (*V*_g_)-drain current (*I*_d_) characteristics.

### 3.3. Fabrication of Fab-Immobilized FET

The silicon dioxide surface of our transistor substrate was exposed to O_2_ plasma (200 W for 1 min) to introduce hydroxyl groups onto the surface, followed by coating with a 3-aminopropyltriethixysilen self-assembled monolayer (APTES SAM). The SAM was formed on the silicon dioxide surface by immersing in 1% (v/w) APTES toluene solution at 60 °C for 7 min in an argon atmosphere. After the SAM modification, the cross-linker, glutaraldehyde (GA), was allowed to react with the amino-terminated surface by immersing the gate area of the APTES-modified FET in a solution of 2.5% GA in 1 × PBS for 30 min. The probe Fab molecules, which were obtained by cleaving the whole anti-AFP antibody, were allowed to react with the aldehyde moiety of the GA-modified surface for 60 min. Subsequently, blocking treatment to avoid non-specific adsorption was performed using EA or BSA. The Fab-immobilized surface was allowed to react with EA (10 mM) or BSA (1 wt%) for 60 min.

### 3.4. Electrical Measurements

The gate voltage-drain current (*V*_g_-*I*_d_) relation of the Fab-immobilized FET was measured and used as a reference. The measurements were made in the dark with a semiconductor parameter analyzer (2612A, Keithley Instruments Inc., Cleveland, OH, USA) at room temperature in 0.01 × PBS (pH 7.4) by sweeping the *V*_g_ from −3 V to 0.5 V with a 0.1 V drain voltage. The reference electrode was Hg/Hg_2_SO_4_. The Fab-immobilized FETs were immersed in the antigen AFP solutions for 60 min. After the immersion, the residue was washed with 0.01 × PBS. The characteristics of *V*_g_-*I*_d_ relation of the antigen-reacted FET was measured in 0.01 × PBS and compared with the reference. The threshold voltage shift (Δ*V*_g_) was then calculated.

### 3.5. Surface Analysis by Using Atomic Force Microscopy

Surface morphologies on the FET surface were analyzed using atomic force microscopy (AFM). The topographic images of the gate surfaces of the FETs were investigated by dynamic mode AFM (SPM-9600, Shimadzu Co., Kyoto, Japan). A silicon cantilever (OMCL-AC240TSC2, Olympus Co., Tokyo, Japan, spring constant 2 N/m, resonance frequency 70 kHz) was used, and the image size was 1 μm × 1 μm with 512 × 512 pixels. Roughness parameters were obtained from the AFM images.

## 4. Conclusions

In this work, we successfully developed a label-free FET biosensor that allows for efficient AFP detection in human serum. To improve the sensitivity to AFP, probe receptors should ideally be small and fixed close to the sensor gate surface. Here, we immobilized small Fab receptors to allow antigen-antibody reactions to occur with the Debye length and improved the sensitivity of the FETs. The Fab-immobilized biosensor was subjected to a blocking treatment to avoid non-specific interactions and showed high-sensitivity and good specificity for AFP in human serum. Our FET biosensor demonstrates good potential as a platform for early clinical diagnosis of tumor markers.
